# Outcomes of kidney‐transplanted patients with history of intestinal reconstruction of the urinary tract

**DOI:** 10.1002/bco2.105

**Published:** 2021-09-03

**Authors:** Juliette Gueguen, Marc‐Olivier Timsit, Anne Scemla, Jean‐Michel Boutin, Franck Bruyere, Hélène Longuet, Rebecca Sberro‐Soussan, Christophe Legendre, Dany Anglicheau, Matthias Büchler

**Affiliations:** ^1^ Department of Nephrology and Clinical Immunology Hospital of Tours Tours France; ^2^ Transplantation, Immunologie, Inflammation (T2I) University of Tours Tours France; ^3^ Department of Urology and Renal Transplantation Georges Pompidou European Hospital, AP‐HP Paris France; ^4^ Paris Cite and Kidney Transplantation Department Necker Hospital, Assistance Publique‐Hôpitaux de Paris, Paris Descartes University Sorbonne Paris France; ^5^ Department of Urology CHU de Tours Tours France

**Keywords:** kidney allograft survival, kidney failure, kidney transplantation, surgical complications, urinary tract deviation, urinary tract dysfunctions, urinary tract malformation

## Abstract

**Background:**

Due to increased risk of pyelonephritis, patients with intestinal reconstruction of the lower urinary tract (IRLUT) have long been advised against kidney transplantation. The aim of this study was to compare the outcomes of transplantation between patients with IRLUT and patients with normal LUT (NLUT) using propensity score matching method.

**Methods:**

The study included 23 kidney recipients with IRLUT matched to 46 kidney recipients with NLUT using known allograft survival and pyelonephritis risk factors as covariates. One‐, 5‐, and 10‐year graft survival, pyelonephritis, and surgical complications occurrence and graft function were compared.

**Results:**

One‐, 5‐, and 10‐year graft survival were 96%, 91%, and 63% in the IRLUT group and 96%, 88%, and 70% in the NLUT group, respectively (*p* = 0.72). Patients with IRLUT had increased cumulative risk of pyelonephritis at 10 years (70% vs. 19%; log‐rank < 0.01) without impacting graft function or rejection occurrence. There was no difference in overall surgical complication, but patients with IRLUT had more urological complications than patients with NLUT (62% vs. 28%; *p* < 0.01).

**Conclusions:**

Our case‐control study consolidates the results regarding the safety of transplantation in patients with IRLUT using a strong validated matching method and provides new insights regarding graft function, pyelonephritis, and surgical complications in this population.

**Abbreviations**
AMR: antibody‐mediated rejection
ATG: antithymocyte globulin
CNIL: Commission Nationale de l'Informatique et des Libertés
DIVAT: Données Informatisées et VAlidées en Transplantation
DSA: anti‐human leucocyte antigen donor‐specific antibodies
ECD: expanded criteria donors
eGFR: estimated glomerular filtration rate
ESRD: end‐stage renal disease
IQR: interquartile range
IRLUT: intestinal reconstruction of the lower urinary tract
MDRD: modification of diet in renal disease
NLUT: normal lower urinary tract
PNA: acute pyelonephritis
SCD: standard criteria donors
TCMR: T cell‐mediated rejection

## 1 INTRODUCTION

Congenital or acquired lower urinary tract (LUT) abnormalities account for approximately 50% of end‐stage renal disease (ESRD) cases in children and 4% of cases in adults, with repetitive pyelonephritis or chronic obstruction being the principle mechanisms.^1^ The primary goal of treatment for patients with LUT abnormalities is to prevent ESRD occurrence, but some patients will unfortunately need to undergo renal replacement therapy. According to some researchers, transplantation in patients with an abnormal LUT has been reported to be associated with an increased risk of pyelonephritis and thus graft loss, whereas other researchers claim that graft survival is comparable between normal and abnormal LUTs, despite patients with LUT abnormalities exhibiting poorer graft function at 10 years.^2^, ^3^, ^4^ Thus, in patients with an abnormal LUT, anatomical, and functional evaluations are recommended to detect and treat abnormalities that may impact kidney allograft survival.^5^ Indeed, when medical treatments, such as patient education, self‐intermittent catheterization, botulin toxin, and/or anticholinergic treatments, are insufficient, surgical intervention becomes the only alternative, leading surgeons to consider transplantation into a reconstructed LUT. The first case of kidney transplantation into a urinary tract deviation with an intestinal conduit was described in 1966 by Kelly et al., followed by multiple short reports with few patients.^6^ Recent studies have reported promising results regarding patient and allograft survival—93% and 87.6%, respectively, at 5 years—despite an increased risk of complications being caused by the presence of intestine in the urinary tract; the prevalence of surgical complications has been reported to be as high as 62%, that of urinary tract infections has been reported to be 83%, and that of metabolic acidosis and an increased risk of lithiasis have been reported to be 70%.^7^, ^8^, ^9^, ^10^, ^11^ These results have been reported in studies conducted in small cohorts or uncontrolled studies that did not adjust for potential risk factors known to impact allograft survival.^11^, ^12^, ^13^, ^14^

Our study aimed to report kidney allograft survival in patients who underwent LUT reconstruction for ESRD due to LUT abnormalities. Kidney transplant recipients who underwent intestinal reconstruction of the LUT (IRLUT) were matched to kidney transplant recipients without LUT abnormalities (NLUT) by the propensity score. We compared patient and graft survival, surgical complications, the occurrence of pyelonephritis, and graft function between the two study groups.

## 2 MATERIALS AND METHODS

### 2.1 Study population

We included 26 consecutively treated patients who previously underwent IRLUT and underwent kidney transplantation between November 1, 2004 and September 1, 2016, at two French centers (Necker Hospital in Paris and Tours University Hospital). Reconstruction involved Bricker or Mitrofanoff deviation, with or without associated enterocystoplasty for malformations or urothelial cancer. We conducted a retrospective case‐control study using propensity score matching. The controls were kidney transplanted patients with NLUTs selected from the Paris kidney transplant cohort.

### 2.2 Clinical data

Data were retrieved from the Données Informatisées et VAlidées en Transplantation (DIVAT) (www.divat.fr) and ASTRE informatized databases. Missing data were individually collected from the patient files. Patients provided written informed consent regarding data collection, data processing, and biopsy. Anonymous data exploitation was performed. Each patient from the present study provided written informed consent to be included in the DIVAT and ASTRE databases. These registries were approved by the National French Commission for bioinformatics data and patient liberty (DIVAT: Commission Nationale de l'Informatique et des Libertés [CNIL], registration number: 1016618, validated June 8, 2004, and ASTRE: CNIL, registration number: DR‐2012‐518).

### 2.3 Transplantation running

The transplantation allocation system was identical for both centers and followed the rules of the French national agency for organ procurement (Agence de la Biomédecine). All transplants were compatible based on the ABO blood group, and negative cytotoxicity cross matching for immunoglobulin G T cell and B cell complements was required for all recipients.

The surgical procedure was conducted by a urologist. Vascular anastomosis was performed with the end‐to‐side technique on external iliac vessels. The type of urological anastomosis achieved depended on the type of reconstruction. Induction therapy involved basiliximab or anti‐thymocyte globulin (ATG), and predominant maintenance therapy involved a calcineurin inhibitor (tacrolimus or cyclosporin), mycophenolic acid, and steroids. Some patients received azathioprine or mammalian targets of rapamycin (mTOR) inhibitor. This treatment was adjusted according to the patient's history of immunization, infectious diseases, and neoplasia. The patients received cotrimoxazole to prevent pneumocystis (for 3 or 6 months or indefinitely, depending on the center's strategy).

The follow‐up included clinical and biological (urine and blood samples) surveillance, which was performed weekly during the first 3 months, twice a month for 3 months, monthly for 1 year, and every 4 months thereafter. Protocol biopsy was performed at 3 and 12 months at Necker Hospital and at 3 months at Tours Hospital. When proteinuria, acute kidney failure, or de novo anti‐human leucocyte antigen donor‐specific antibodies (DSAs) were detected, biopsies were performed at both centers. Rejection was classified according to the corresponding period Banff classification.^15^, ^16^

### 2.4 Evaluation criteria

The primary endpoint was kidney allograft survival. Allograft survival was defined by a definitive return to dialysis or repeated transplantation. The secondary endpoints included patient survival, kidney function, surgical complications, pyelonephritis, and the incidence of rejection. The glomerular filtration rate (eGFR) was estimated using the Modification of Diet in Renal Disease (MDRD) formula. Delayed graft function was defined as the need for dialysis immediately after transplantation. The surgical complications included events potentially linked to the transplant surgery and/or vascular or LUT reconstruction: vascular complications (hematoma, arterial or venous thrombosis, aneurysm, and arterial stenosis), urological complications (urinoma, compressive lymphocele, reflux, ureteral stenosis, stomal, or conduit stenosis), or intestinal complications (occlusion and peritoneal wound). Only surgical complications classified ≥2 according to Clavien and Dindo were collected.^17^ Pyelonephritis was defined by the presence of pathogenic germs in the urine associated with fever and requiring hospitalization and antibiotics. Rejections were classified according to the corresponding period Banff classification.^15^, ^16^

### 2.5 Statistical analysis

We used the propensity score to match patients who underwent IRLUT to patients with a NLUT. The propensity score was estimated using a logistic regression model predicting a history of reconstruction with the following predictive covariates: recipient age and sex, donor age, donor type (extended criteria donor [ECD], standard criteria donor [SCD], or living donor), transplant rank, preformed DSA, induction therapy, and a history of diabetes. Matching was performed with log‐transformed propensity scores to approximate a normal distribution. Matching was performed with the nearest‐neighbor method without replacement using R (version 3.2.1, R foundation for statistical computing). The NLUT individuals were matched to IRLUT patients with a 2:1 ratio and a caliper width of 0.3. Continuous variables were described using medians and interquartile ranges. Qualitative variables were expressed as percentages (%). Continuous variables were compared with the Mann–Whitney test, and qualitative variables were compared with Fisher's exact test. Death‐censored graft survival and patient survival were assessed from the time of transplantation to a maximum follow‐up of 10 years using Kaplan–Meier curves and were compared using the log‐rank test. Pyelonephritis occurrence was assessed from the time of transplantation to a maximum follow‐up of 10 years using cumulative incidence curves and was compared using the log‐rank test. A *p* value of <0.05 was considered significant.

## 3 RESULTS

### 3.1 Baseline characteristics in patients with IRLUT

Twenty‐six patients who underwent IRLUT were included in the study. Their characteristics are presented in Table 1. LUT abnormalities consisted of 16 (62%) LUT malformations (two cases of Prune‐Belly syndrome, five cases of posterior urethral valves, two cases of reflux, and seven undefined cases), six (23%) LUT dysfunctions (five cases of spina bifida and 1 undefined case), and four (15%) urothelial carcinomas that were treated with cystectomies. The patients underwent IRLUT at a median age of 19 years old with a median time between reconstruction and transplantation of 31 months. The median recipient age at transplantation was 28 years old. Of note, the patients with urothelial cancer underwent transplantation at a median age of 52 years, whereas the patients with malformations underwent transplantation at younger age, with a median age of 27.5 years. Reconstruction involved Bricker deviation in 10 (38%) cases, Mitrofanoff deviation in three (12%) cases, enterocystoplasty in six (23%) cases, and enterocystoplasty (for neobladder or for) bladder augmentation) associated with a conduit in seven (27%) cases. For two patients, the second transplantation surgery was considered in the study because of the inclusion time. The first patient lost his first graft at Day 0 because of immediate thrombosis, and the second lost his graft because of repetitive infections (data unavailable, foreign country). Four patients had pre‐existing DSA. One patient had diabetes. The median donor age was 30 years old, including four (15.4%) living donors. All patients underwent induction therapy, and depletant induction with ATG was administered in 6 (26%) patients. Twenty‐two (85%) patients underwent maintenance therapy including a CNI and mycophenolic acid, and 23 (88%) were administered long course steroids. The median cold ischemia time was 1001 min. Seventeen (74%) patients received double or mono‐J stenting for a median time of 26.5 days.

**TABLE 1:** Characteristics and pyelonephritis episodes of patients with lower urinary tract (LUT) reconstruction (IRLUT), *N* = 26

Patient	Sex	ESRD cause	Malformation type	Age at reconstruction (years)	Type of reconstruction	Age at transplantation (years)	Donor age (years)	Type of anastomosis	Surgical complications	PNA (*n*)	Self‐intermittent catheterization (Y/N)	Time of follow‐up after transplantation (years)
1	F	Malformative	Reflux	27	Bricker	28	25	Uretero‐conduit		0	N	3
2	M	Malformative		<1	Bricker	45	48	Uretero‐ureteral		0	N	12
3	M	Malformative		19	Bricker	26	49	Uretero‐conduit	Bricker stenosis	0	N	8
4	M	Urothelial carcinoma		56	Bricker	58	53	Uretero‐conduit	Occlusion	1	N	5
5	F	Neurogenic bladder	Spina bifida	25	Bricker	27	23	Uretero‐conduit	Obstructive urethritis	≧2	N	6
6	M	Malformative	Prune‐Belly	9	Bricker	9	24	N/A	Bricker perforation; Lithiasis	≧2	N	14
7	M	Malformative	Prune‐Belly + reflux+ posterior urethral valves	4	Bricker	19	29	Uretero‐ureteral		≧2	N	4
8	M	Urothelial carcinoma		75	Bricker	75	69	Uretero‐conduit	Lymphocele; GAS; Lithiasis	≧2	N	2
9	F	Malformative		57	Bricker	59	57	N/A	Peritoneal wound	N/A		0
10	M	Neurogenic bladder	Spina bifida	13	Bricker (colon)	33	19	Uretero‐conduit	Obstructive pyelonephritis	≧2	N	6
11	F	Neurogenic bladder	Spina bifida	31	Enterocystoplasty	40	68	Uretero‐ureteral		≧2	Y	3
12	M	Neurogenic bladder	Spina bifida	38	Enterocystoplasty	43	31	Uretero‐ureteral	Venous thrombosis	≧2	Y	6
13	F	Malformative		3	Enterocystoplasty	28	40	N/A	GAS	0	Y	11
14	M	Urothelial carcinoma		50	Enterocystoplasty	56	50	Uretero‐neobladder	GAS	1	N	3
15	M	Urothelial carcinoma		65	Enterocystoplasty	66	63	Uretero‐neobladder		1	N/A	1
16	M	Malformative	Posterior urethral valves	13	Enterocystoplasty + Mitrofanoff	16	12	Uretero‐neobladder	Reflux	≧2	Y	9
17	M	Malformative		11	Enterocystoplasty + Mitrofanoff	17	14	Uretero‐neobladder		0	Y	2
18	M	Malformative	Posterior urethral valves	6	Enterocystoplasty + Mitrofanoff	7	8	Uretero‐ureteral	Urinoma; Reflux	1	Y	13
19	M	Malformative	Posterior urethral valves	26	Enterocystoplasty + Mitrofanoff	27	23	Uretero‐neobladder	Incontinence	≧2	Y	2
20	F	Malformative		20	Enterocystoplasty + Mitrofanoff	23	30	Uretero‐neobladder		≧2	Y	1
21	F	Neurogenic bladder	Spina bifida	12	Enterocystoplasty + Mitrofanoff	13	14	Uretero‐neobladder	Lithiasis	≧2	Y	12
22	M	Malformative	Posterior urethral valves	4	Gastrocystoplasty	29	25	Uretero‐ureteral		0	Y	2
23	F	Malformative		19	Gastrocystoplasty + Bricker	25	45	Uretero‐ureteral	Reflux	N/A	N	13
24	M	Malformative	Posterior urethral valves	17	Mitrofanoff	18	5	Uretero‐bladder		≧2	Y	2
25	M	Neurogenic bladder		26	Mitrofanoff	39	45	Uretero‐bladder		0	Y	4
26	M	Malformative	Reflux	14	Mitrofanoff	17	15	Uretero‐bladder	Venous thrombosis	0	Y	5

Abbreviations: ECD, expanded criteria deceased donor; F, female; GAS, graft arterial stenosis; LD, living donor; M, male; N, no; PNA,posttransplant pyelonephritis; SCD, standard criteria deceased donor; Y, yes.

### 3.2 Baseline characteristics of patients with a NLUT

Patients #16, #17, and #24 could not be matched according to the propensity score, so that we were able to match 23 IRLUT patients to 46 NLUT patients using the propensity scores and the criteria described in Section 2 (Table 2). The causes of ESRD included glomerulopathy in 19 (41%) patients, diabetic nephropathy in five (11%) patients, interstitial nephropathy in six (13%) patients, polycystic kidney disease in one (2%) patient, other causes in six (13%) patients, and conditions without a known etiology in 10 (22%) patients. For six patients, the second transplantation was considered for this study. Fifteen (58%) patients received double J stenting.

**TABLE 2:** Characteristics of the patients who underwent lower urinary tract (LUT) reconstruction (IRLUT) and patients with a normal LUT (NLUT) after matching

Baseline characteristics	IRLUT (*n* = 23)	NLUT (*n* = 46)	*p*
Median recipient age (IGR)	28 (24–44)	39 (22–54)	0.32
Males, *n* (%)	15 (65)	21 (59)	0.79
Pre‐existing diabetes (%)	1 (4)	1 (2)	1
Pre‐existing DSA (%)	4 (17)	6 (13)	0.72
Living donors (%)	4 (17)	5 (11)	0.47
Extended criteria donors (%)	2 (9)	14 (34)	0.12
Median donor age (IGR)	31 (24–50)	49 (21–59)	0.31
ATG induction (%)	6 (26)	25 (57)	0.02
Median cold ischemia time (min) (IQR)	1175 (871–1560)	1146 (772–1608)	0.98

Abbreviations: ATG, anti‐thymocyte globulin; DSA, donor‐specific antibody; IQR, interquartile range.

### 3.3 Death‐censored kidney allograft survival

**FIGURE 1:**
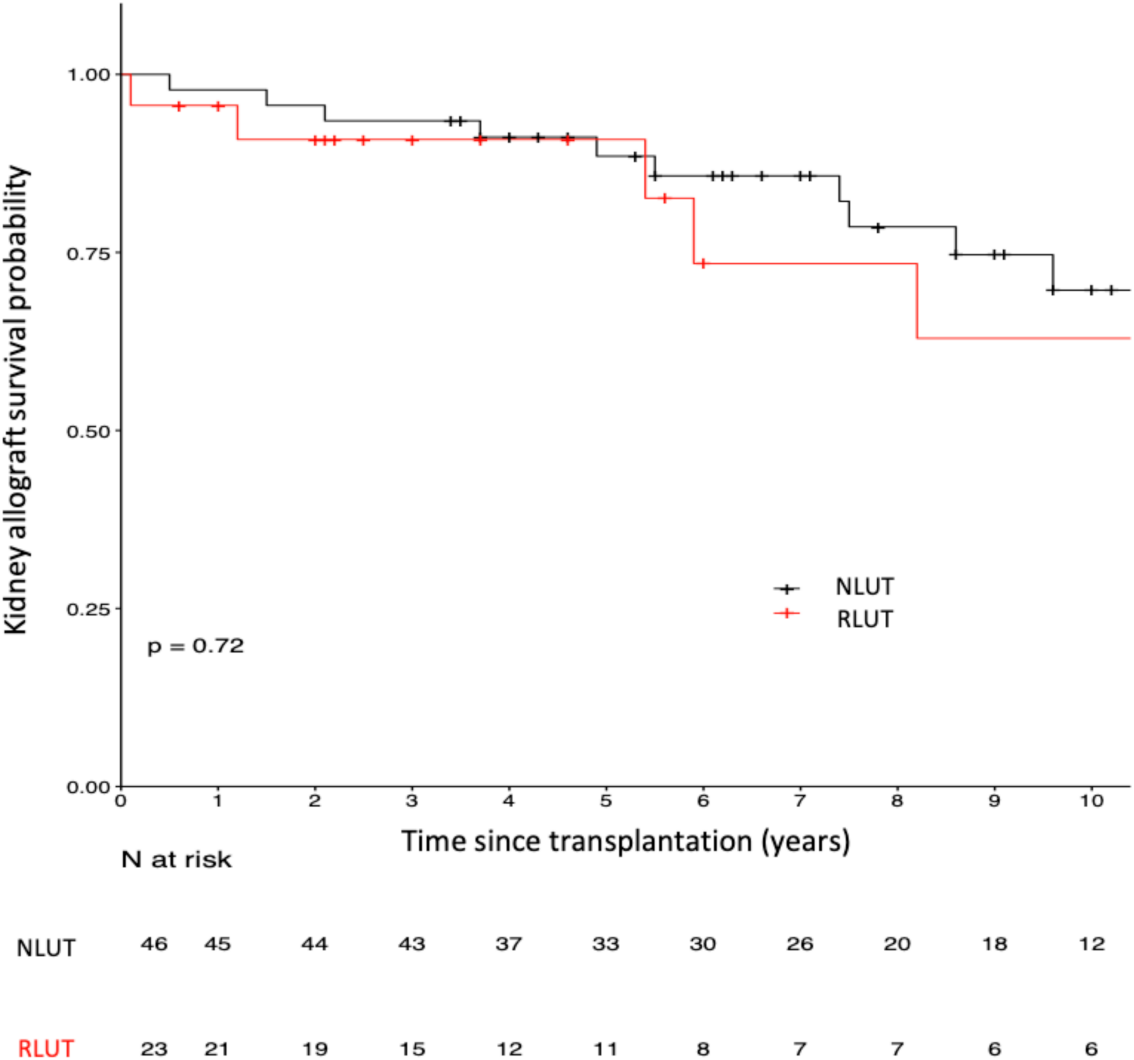
Death‐censored allograft survival curves in patients who underwent lower urinary tract (LUT) reconstruction (IRLUT) and matched controls with a normal LUT (NLUT)

The mean follow‐up time was 6.81 years, and the graft survival rates at 1, 5, and 10 years were 96%, 91%, and 63% in the IRLUT group and 96%, 91%, and 70% in the NLUT group, respectively (*p* = 0.72, Figure 1). In the IRLUT group, five grafts were lost due to rejection (*n* = 2), pyelonephritis (*n* = 1), and undefined causes (*n* = 2). In the NLUT group, 11 grafts were lost due to rejection (*n* = 6), urological complications (*n* = 1), and undefined causes (*n* = 4).

### 3.4 Overall patient survival

**FIGURE 2:**
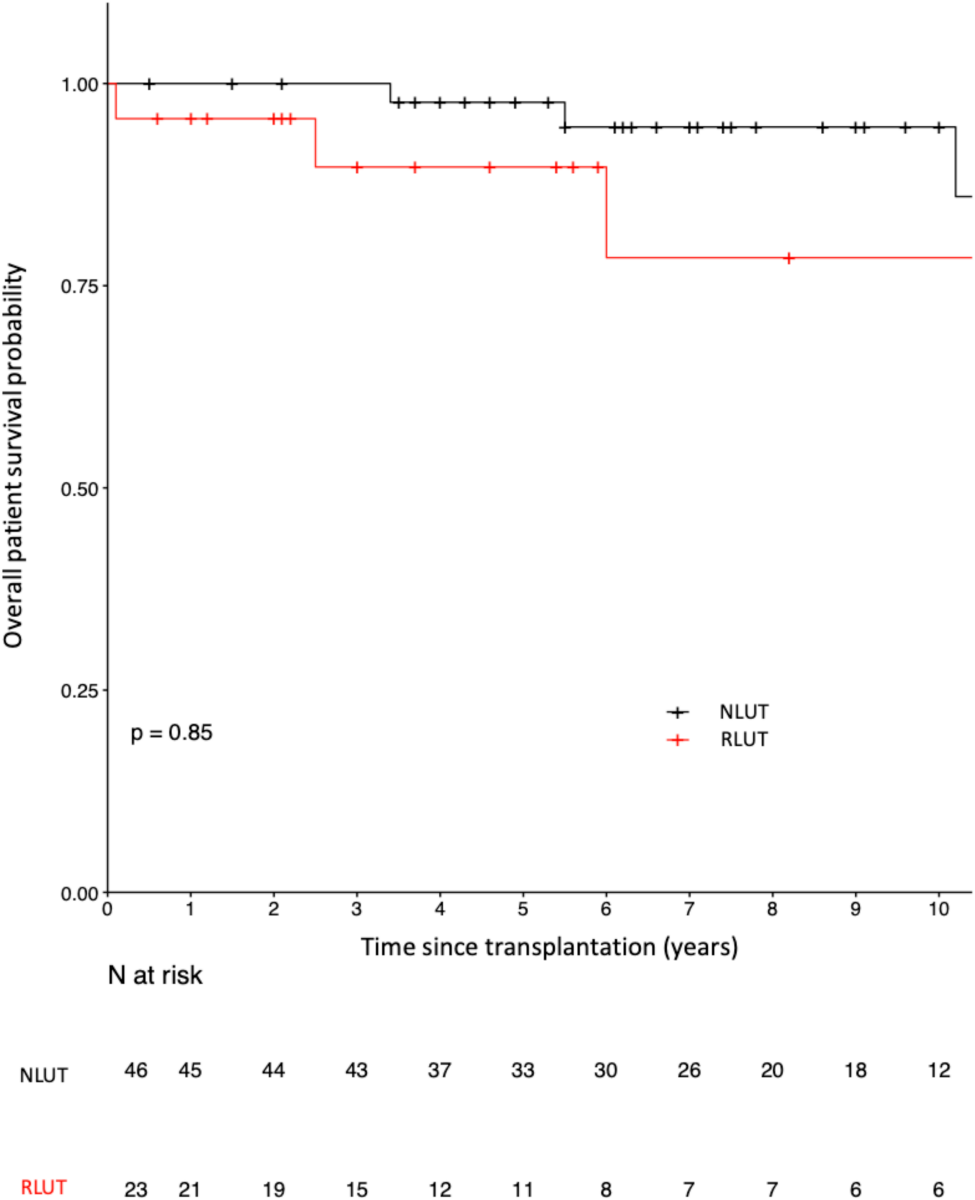
Patient survival curves in the patients who underwent lower urinary tract (LUT) reconstruction (IRLUT) and matched controls with a normal LUT (NLUT)

The patient survival rates at 1, 5, and 10 years were 96%, 90%, and 79% in the IRLUT group and 100%, 98%, and 95% in the NLUT group, respectively (*p* = 0.9, Figure 2). In the IRLUT group, three patients died from extraurinary neoplasia (*n* = 1) and undefined causes (*n* = 2). In the NLUT group, seven patients died from septicemia (*n* = 2), ESRD (*n* = 1), cardiovascular conditions (*n* = 1), and undefined causes (*n* = 3).

### 3.5 Pyelonephritis episodes

**FIGURE 3:**
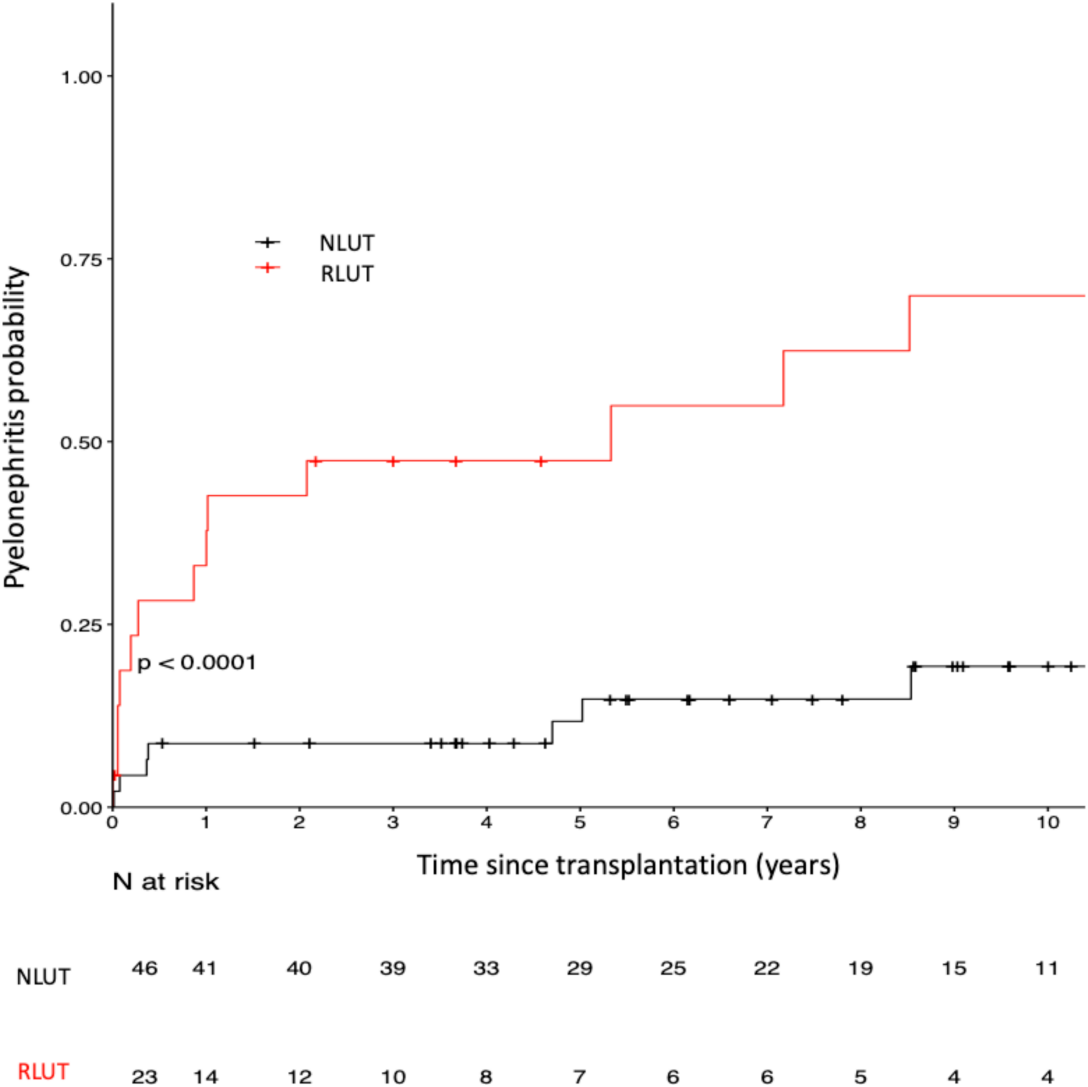
Pyelonephritis occurrence in patients who underwent lower urinary tract (LUT) reconstruction (IRLUT) and matched controls with a normal LUT (NLUT)

More patients in the IRLUT group than in the NLUT group had at least one episode of pyelonephritis (65% vs. 17%, *p* < 0.01). The probability of having pyelonephritis at 10 years was 70% in the patients who underwent IRLUT and 19% in the patients with a NLUT (log‐rank < 0.01) (Figure 3). In the IRLUT group, 15 (65%) patients had at least 1 case of pyelonephritis, 13 (86%) of whom had recurrent pyelonephritis, and the median time from transplantation to the first case of pyelonephritis was 317 days. In the NLUT group, eight (17%) patients had at least one case of pyelonephritis, of whom four (50%) had recurrent pyelonephritis, and the median time from transplantation to the first case of pyelonephritis was 924 days. Of note, 96% of patients in the IRLUT group had chronic persistent bacteriuria, and 33% had persistent multidrug‐resistant bacteria.

### 3.6 Surgical complications

In the IRLUT group, 15 (65%) patients had 20 adverse events, 62% of which had a urological origin, 10% of which had an intestinal origin, and 28% of which had a vascular origin. The adverse events were as follows: four cases of urolithiasis, one case of compressive lymphocele, one case of urinoma, two cases of reflux, one case of incontinence, two obstructive urinary tract infections, one case of Bricker perforation, one case of Bricker stenosis, one peritoneal wound, one occlusion, and six arterial or venous complications. Nineteen (95%) of these cases required surgical revision (Grade III). No Grade IV or V complications were identified.

In the NLUT group, 25 (54%) patients had 32 adverse events, 28% of which had a urological origin and 72% of which had a vascular origin. The adverse events were as follows: four cases of lymphoceles, two cases of urinoma, one case of reflux, two cases of ureteral stenosis, eight cases of hematoma, and 15 cases of arterial or venous complications, including nine arterial stenoses. Eleven (34%) of these cases required surgical revision (Grade III). No grade IV or V complications were identified. The patients who underwent IRLUT had more urological complications (62% vs. 28%, *p* < 0.01), specifically more lithiasis (19% vs. 0%, *p* = 0.01) and fewer vascular complications (28% vs. 72%, *p* < 0.01), than did the patients with a NLUT (Table 3A).

**TABLE 3:** Posttransplantation Outcomes in patients who underwent lower urinary tract (LUT) reconstruction (IRLUT) and in matched patients with a normal LUT (NLUT)

A: Surgical complications occurring in patients who underwent LUT reconstruction (IRLUT) and in matched patients with a normal LUT (NLUT)					
Complications		IRLUT (*n* = 23)	NLUT (*n* = 46)		*p*
Patients with surgical complications, *n* (%)		15 (65)	25 (54)		0.45
Surgical complications, *n*		21	32		NS
Urological complications, *n* (%)		13 (62)	9 (28)		<0.01
Lithiasis, *n* (%)		4 (19)	0 (0)		0.01
Lymphocele, *n* (%)		1 (5)	4 (12,5)		NS
Urinoma, *n* (%)		1 (5)	2 (7)		NS
Reflux, *n* (%)		2 (9)	1 (3)		NS
Incontinence, *n* (%)		1 (5)	0 (0)		NS
Ureteral stenosis, *n* (%)		0 (0)	2 (7)		NS
Obstructive urethritis, *n* (%)		1 (5)	0 (0)		NS
Obstructive pyelonephritis, *n* (%)		1 (5)	0 (0)		NS
Bricker stenosis/perforation, *n* (%)		2 (9)	‐		‐
Intestinal complications, *n* (%)		2 (10)	0 (0)		NS
Peritoneal wound, *n* (%)		1 (5)	0 (0)		NS
Occlusion, *n* (%)		1 (5)	0 (0)		NS
Vascular complications, *n* (%)		6 (28)	23 (72)		<0,01
Thrombosis/stenosis/fistula, *n* (%)		6 (28)	15 (47)		NS
Hematoma, *n* (%)		0 (0)	8 (25)		0.05

B: Allograft function at 3 months, 1 year, 5 years, and 10 years in patients who underwent LUT reconstruction and in matched controls with a normal LUT					
Time from transplantation	IRLUT (*n* = 23)		NLUT (*n* = 46)		*p*
	*N*	eGFR (ml/min)	*N*	eGFR (ml/min)	
3 months	22	68 (47–95)	42	61 (47–78)	0.41
1 year	22	59 (48–89)	40	58 (43–72)	0.37
5 years	10	73 (42–83)	32	57 (41–77)	0.59
10 years	6	79 (49–85)	11	59 (36–62)	0.10

Abbreviation: eGFR, estimated glomerular filtration rate using the MDRD formula.

### 3.7 Graft function

Delayed graft function was observed in 5/23 (22%) patients in the IRLUT group and in 13/45 (31%) in the NLUT group (*p* = 0.78). Kidney allograft function was determined by eGFR at 1, 5, and 10 years after transplantation, and the values were similar between patients who underwent IRLUT and patients with a NLUT, reaching 79 and 59 ml/min, respectively, at 10 years (Table 3B).

### 3.8 Rejection

No significant difference between the two study groups was observed in the incidence of rejection episodes (*p* = 0.43). Seven (30%) patients who underwent IRLUT had a rejection episode at a median time of 3 months: four borderline cases of acute T cell‐mediated rejection (TCMR), one case of acute TCMR, one case of antibody‐mediated rejection (AMR), and one case of mixed rejection. Twenty (43%) patients in the NLUT group had a rejection episode at a median time of 3 months: four borderline cases of acute TCMR, nine cases of TCMR, two cases of AMR, and five cases of mixed rejection.

### 3.9 Other complications

In the IRLUT group, no patients developed urinary tract neoplasia at a mean follow‐up of 5.6 years. Of note, no recurrence occurred in patients with previous urothelial carcinoma at a mean follow‐up of 2.3 years.

In the IRLUT group, 12 patients (33%) developed metabolic acidosis requiring steady supplementation with a median dose of 3 g of bicarbonates per day.

## 4 DISCUSSION

Intestinal reconstruction of the LUT is required for cases of acquired or congenital LUT abnormalities when medical treatment is ineffective. This surgery is mostly required in children with congenital malformations and adults with a history of pelvic cancer. Due to its simplicity, Bricker deviation remains the most commonly performed technique, but continent deviation through cystoplasties with or without cutaneous deviation was developed to preserve patients' body images. The goal of treatment is to maintain low pressure in the urinary tract system to prevent complications such as infection and lithiasis and delay the onset of ESRD. Unfortunately, for some patients, renal replacement therapy is mandatory. For most of these patients, kidney transplantation remains the best option in terms of cost, life survival, and quality of life.^18^, ^19^, ^20^ Patients should be aware that there is an increased risk of complications with transplantation into a dysfunctional LUT. Indeed, patients with an underived dysfunctional LUT have been shown to exhibit poor outcomes after transplantation. Thus, pretransplant or peritransplant surgery to correct LUT remains the safest option for successful transplantation.^4^, ^21^, ^22^ The optimal interval between reconstruction and transplantation has been debated, but most of the authors have recommended reconstruction before transplantation for improved patient education, bladder capacity with a saline physiologic solution and healing.^7^, ^14^, ^23^, ^24^ In our study, the median time between reconstruction and kidney transplantation was 31 months, which is longer than the interval described in previous studies, probably because the diagnosis and therapeutic strategies concerning LUT abnormalities have evolved over the past decades; moreover, the strategy probably should differ according to the time spent in dialysis and the patient's bladder capacity. We do not have enough information to answer this question. Because our aim was to study intestinal reconstruction of the LUT as an independent predictor for allograft loss, we decided to include all LUT reconstructions using intestinal segments, regardless of the indication (cancer or malformation) or reconstruction type although most of the studies included primarily LUT malformations or dysfunctions (Table S1). The robustness of our study relies on propensity score matching, which provides adjustment on prognosis factors recognized for influencing graft loss and long‐term outcomes. The involvement of two centers with a high volume of adult (*N* = 342/year) and pediatric (*N* = 26/year) transplantation cases permits a large representation of LUT pathologies despite their scarcity.^1^ Moreover, the 6‐year follow‐up with a short period of inclusion improves the robustness of our results. The restrictive and recent inclusion time ensures uniformity in the management of patients who underwent transplantation. This case‐control study provides reliable results regarding the safety of performing transplantation in patients who have undergone IRLUT, and the allograft‐patient survival and graft function at 10 years were similar between these patients and the matched patients with NLUT. We confirm that patients who have undergone IRLUT have an increased risk of pyelonephritis with high risk of recurrence and give new insights regarding graft function and rejection episodes; moreover, we studied complications in great detail. Indeed, despite the increased number of episodes of pyelonephritis, the patients who underwent IRLUT did not show worse kidney function or more rejection episodes than did the matched patients with NLUT. These results can be explained by a close follow‐up, patient education on the signs, and symptoms indicating a need for hospitalization and allowing early patient care.^25^ In addition to the increased risk of pyelonephritis, patients who underwent IRLUT exhibited more cases of lithiasis. This issue, which has been reported in previous studies, relies on the intestinal segment for several reasons: urinary tract colonization, mucus secretion, hypercalciuria secondary to metabolic acidosis, and urine stagnation in the bladder.^26^ This issue highlights the need for a close follow‐up with a high liquid intake volume, diet education, and control for acidosis. The risk of vascular complications was reported to be lower in the LUT group than in the control group in our study, which probably reflects the experience of the surgeons requested for such procedures. To strictly compare the outcomes regarding surgical complications, it would have been interesting to assess surgeon experience and incorporate it into the propensity score. However, we assume that the immediate development of surgical complications would not have impacted allograft survival, allograft function, or rejections in any way. Despite the several strengths previously reported, our study includes some limitations. First, results need to be interpreted regarding small sample size because we deal with a scarce pathology. Secondly, the propensity score matching is constructed according to the covariates we estimated to be the most appropriate to assess allograft loss risk and urinary infection risk. Because this score is constructed on a logistic regression model, the number of covariates is limited to allow convergence, to be able to keep a reasonable sample size and to obtain estimates with a minimal noise. Some other cofounding variables regarding allograft survival might thus be missing and induce interpretation bias. We tried to include in the score the most relevant ones so that our results remain interpretable.

To confirm our findings and expand our knowledge, our results should be confirmed by other studies, and studies should focus on identifying risk factors associated with poor outcomes using larger cohorts. Surgical techniques regarding the type of reconstruction, urological anastomosis and medical strategy regarding infection prophylaxis, and managing acidosis should be studied in detail.

## 5 CONCLUSION

Our results confirm that renal transplantation is safe in patients who have undergone intestinal reconstruction of the low urinary tract in comparison to matched patients with a low urinary tract with normal function. However, this population has an increased risk of several complications warranting expert management and close follow‐ups.

## FUNDING INFORMATION

The authors declare no funding was received for this study.

## CONFLICT OF INTEREST

The authors declare no conflicts of interest.

## AUTHOR CONTRIBUTION

Juliette Gueguen participated in research design, in the writing of the paper, and in data analysis. Marc‐Olivier Timsit participated in research design and in the writing of the paper. Anne Scemla participated in research design and in the writing of the paper. Jean‐Michel Boutin participated in the writing of the paper. Franck Bruyère participated in the writing of the paper. Hélène Longuet participated in the writing of the paper. Rebecca Sberro‐Soussan participated in the writing of the paper. Christophe Legendre participated in the writing of the paper. Dany Anglicheau participated in research design and in the writing of the paper. Matthias Büchler participated in research design and in the writing of the paper.

## Supporting information


**Table S1:** Literature review about transplantation in patients who underwent intestinal reconstruction of the LUT
