# Supplementary material for: Outcomes of kidney‐transplanted patients with history of intestinal reconstruction of the urinary tract
Source: BJUI Compass. 2021 Sep 3;3(1):75–85. doi: 10.1002/bco2.105 (PMC8988834; doi:10.1002/bco2.105)
Supplement: Supplementary file 1 — Table S1: Literature review about transplantation in patients who underwent intestinal reconstruction of the LUT [file BCO2-3-75-s001.docx]

Supplementary - Table 1: Literature review about transplantation in patients who underwent intestinal reconstruction of the LUT

| Author | | Journal | Study type | Year of publication | N | Inclusion period | Median follow up time (years) | Type of reconstruction | Median age at Tx | Graft survival | Pyelonephritis | Surgical complications |
| --- | --- | --- | --- | --- | --- | --- | --- | --- | --- | --- | --- | --- |
| Glass | Journal of Urology | | Review | 1985 | 68 |  |  | Incontinent cutaneous diversions |  |  | 19% (mainly associated with stenosis or lithiasis) leading to death in 70% cases | 32%; due to construction |
| Fontaine | Journal of Urology | | Cohort | 1998 | 14 | 1972-1996 | 6,6 | Enterocystoplasty | 12 | 84% 5Y | 28% | 14% lithiasis |
| Warholm | Nephrology, Dialysis, Transplantation | | Controlled | 1999 | 27 | 1982-1996 |  | Continent and incontinent cutaneous diversion | 40 | 90% 1Y 70% 5Y | 1 PNA  100% chronic asymptomatic bacteriuria | Longer procedures |
| Surange | Journal of Urology | | Cohort | 2003 | 54 | 1980-2001 | 4,6 | Incontinent diversion | 28 | 90% à 1Y 63% à 5Y 52% à 10Y | 65%  50% recurrence | 47% including 60% due to reconstruction  (Lithiasis, anastomosis stenosis, obstruction, occlusion, stomal hernia, stomal stenosis) |
| Hatch | Journal of Urology | | Cohort | 2001 | 31 |  |  | Incontinent and continent cutaneous diversions Enterocystoplasty | 12 | 90% 1Y 78% 5Y 60% 10Y | 69% | 23% (stomal stenosis, entero-bladder fistula, lithiasis, hernia, ureteral stenosis, wound dehiscence) |
| Fournier | International Journal of Urology | | Cohort | 2017 | 16 | 1987-2010 | 14 | Continent cutaneous diversions | 37 | 73% 10Y 66% 15Y | 50% | 50% (6 reinterventions on reconstruction, 2 graft arterial stenosis, 1 lymphocele)  37% lithiasis |

N= number of patients, Y= year(s); PNA= pyelonephritis
